# Reversible Ferroelectric Polarization Modulation of Chiral Molecular Ferroelectrics by Circularly Polarized Light

**DOI:** 10.1002/advs.202414977

**Published:** 2025-01-21

**Authors:** Zhongxuan Wang, Qian Wang, Lina Quan, Shenqiang Ren

**Affiliations:** ^1^ Department of Materials Science and Engineering University of Maryland College Park MD 20742 USA; ^2^ Department of Chemistry Virginia Tech Blacksburg Blacksburg VA 24060 USA; ^3^ Department of Materials and Science Engineering Virginia Tech Blacksburg Blacksburg VA 24060 USA

**Keywords:** axial chirality, chiral ferroelectricity, CPL modulation, reversible polarization, thermal conductivity modulation

## Abstract

The optical modulation of ferroelectric polarization constitutes a transformative, non‐contact strategy for the precise manipulation of ferroelectric properties, heralding advancements in optically stimulated ferroelectric devices. Despite its potential, progress in this domain is constrained by material limitations and the intricate nature of the underlying mechanisms. Recent studies have achieved efficient regulation of ferroelectric polarization and thermal conductivity in chiral ferroelectric thin films through the application of left‐ and right‐handed circularly polarized light (LCP and RCP). Differential absorption of circularly polarized light (CPL) induces nonequilibrium carrier dynamics, generating distinctive interfacial electrostatic fields that enable precise control of ultrathin ferroelectric films. For (R)‐BINOL−DIPASi and (S)‐BINOL−DIPASi (C_26_H_26_O_2_Si), polarization changes surpass 23%, exhibiting opposite response under LCP and RCP excitation. In R chiral films, remnant polarization decreases from 1.05 µC cm^−^^2^ under LCP to 0.85 µC cm^−^^2^ under RCP, whereas in S chiral films, polarization increases from 0.85 µC cm^−^^2^ under LCP to 0.98 µC cm^−^^2^ under RCP. This reversible modulation facilitates reliable switching between ON and OFF states, presenting the potential of chiral ferroelectric materials for flexible, high‐speed integrated photonic sensor technologies.

## Graphical abstract

Chiral ferroelectric thin films exhibit tunable ferroelectric polarization and thermal conductivity under CPL. Differential absorption generates distinct interfacial electrostatic fields, enabling reversible modulation of polarization. This discovery highlights the potential of chiral ferroelectric materials for high‐speed integrated photonic devices, with remnant polarization changes exceeding 23% under CPL excitation.

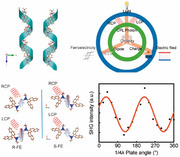

## 1 Introduction

The modulation of ferroelectric polarization is a pivotal technique for enabling the functional applications of ferroelectric materials in non‐volatile memory devices,^[^ ^1^, ^2^ ^]^ electro‐optic modulators,^[^ ^3^, ^4^ ^]^ piezoelectric devices,^[^ ^3^, ^5^ ^]^ and pyroelectric sensors.^[^ ^6^, ^7^ ^]^ Currently, static or pulsed electric fields and strain at heterojunction interfaces are widely regarded as the most effective methods for polarization control. However, electric field‐based modulation requires circuit integration and involves complex operational processes, while strain engineering in artificial heterojunction structures requires high‐precision epitaxial techniques to address lattice mismatch constraints.^[^ ^8^, ^9^ ^]^ Moreover, electric field control is inherently limited by slow switching speeds and circuit dependence, rendering it insufficient for achieving ultrafast polarization switching.^[^ ^10^, ^11^ ^]^ Similarly, mechanical strain cannot facilitate ultrafast dynamic modulation of ferroelectric polarization.^[^ ^12^ ^]^ To overcome these challenges in ferroelectric polarization control, researchers have increasingly focused on employing optical strategies to achieve precise modulation. This approach not only introduces novel degrees of freedom but also demonstrates significant potential for advancing high‐speed ferroelectric devices. In recent years, reversible optical control of ferroelectric polarization has become increasingly feasible. This breakthrough enables ferroelectric devices to transcend the limitations of circuit dependence and interface engineering, allowing for non‐contact and remote operation, thereby paving the way for the functional development of next‐generation ferroelectric materials.^[^ ^13^, ^14^ ^]^ Compared to the modulation of ferroelectric polarization achieved through local thermal effects induced by high‐power lasers, polarization switching driven by photoinduced non‐equilibrium carriers requires significantly lower illumination energy densities. Specifically, the power density required for polarization reversal through photoinduced carrier effects is more than five orders of magnitude lower than that required for polarization switching induced by high‐power laser irradiation.^[^ ^14^, ^15^ ^]^ An important parameter in the optical control of ferroelectric polarization is the polarization state of the light, which provides a new degree of freedom beyond wavelength and light intensity.^[^ ^16^ ^,^ ^19^ ^]^ Compared to linearly polarized light, CPL can achieve precise control of polarization in ferroelectric material by altering the left‐handed and right‐handed polarization states, without considering the polarization direction or orientation of ferroelectric crystal. Consequently, the CPL control of polarization in ferroelectric materials is highly attractive for applications in ultrafast, low‐energy, non‐contact integrated optical sensors, data storage, and logic devices.^[^ ^20^ ^,^ ^23^ ^]^ Although enhancing symmetry breaking has enabled the regulation of circularly polarized photons by electric fields in the study of coupling effects between circularly polarized photons, electric fields, and ferroelectric order.^[^ ^24^ ^,^ ^27^ ^]^ However, the reverse process, namely the manipulation of electric dipole polarization through circularly polarized photons, has not yet been achieved.^[^ ^13^, ^28^ ^]^ Due to possessing both ferroelectricity and optical activity, molecular chiral ferroelectrics offer an effective pathway for controlling polarization with circularly polarized photon, introducing new degrees of freedom and coupling mechanisms.^[^ ^29^ ^,^ ^35^ ^]^ Because of their unique mirror‐asymmetric structures, molecular chiral materials exhibit unique responses to RCP and LCP.^[^ ^36^ ^,^ ^39^ ^]^

In this study, we report the CPL modulation of ferroelectric polarization in molecular chiral ferroelectrics. The different absorption rates of left‐ and right‐handed CPL by chiral ferroelectrics generate varying amounts of nonequilibrium carriers that accumulate at the interface between ferroelectric layer and electrodes, causing electrostatic changes. This enables the modulation of ferroelectric polarization by circularly polarized photons. Consequently, (R)‐BINOL−DIPASi (R‐FE) and (S)‐BINOL−DIPASi (S‐FE) chiral ferroelectric crystals exhibit opposite polarization phenomena when exposed to LCP and RCP. For R chiral ferroelectrics, the remnant polarization decreases from ≈1.05 to 0.85 μc cm^−2^ when the excitation light switches from LCP to RCP. In contrast, for S chiral ferroelectrics, the remnant polarization under LCP excitation is 0.85 μc cm^−2^, significantly lower than that of under RCP (0.98 μc cm^−2^). Moreover, the modulation of polarization by CPL is a reversible process. Additionally, the polarization change induced by photogenerated nonequilibrium carriers leads to dipole flipping, increasing the photogenerated carrier transport barrier, resulting in the decreased current and increased resistance. This enables the CPL photons to switch ferroelectric devices between ON and OFF states. This further confirms the CPL photon modulation of polarization in chiral ferroelectrics and provides potential applications for flexible high‐speed integrated photonic sensor devices.

## 2 Results

**Figure 1:**
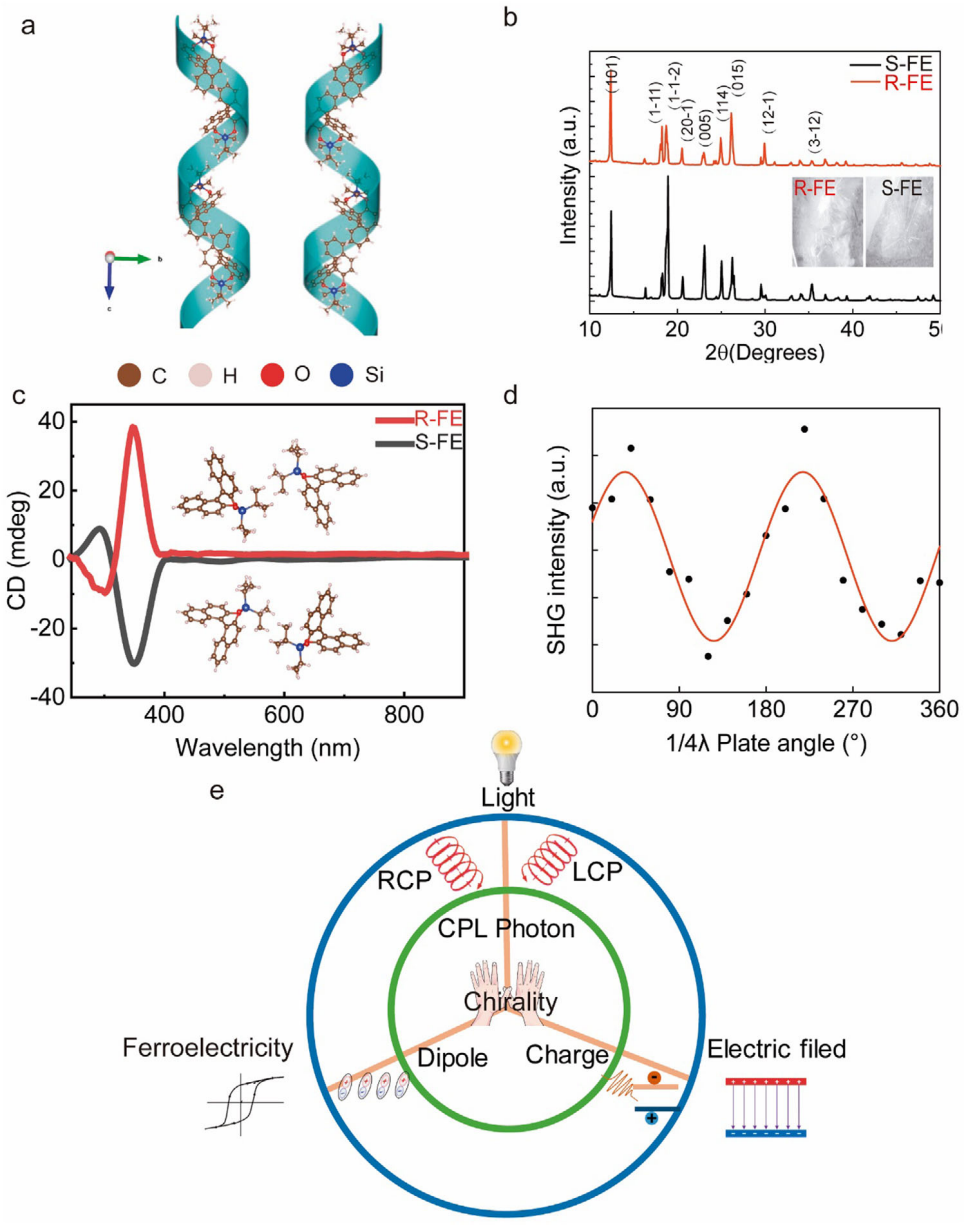
a) The schematic diagram of the helical axis chiral structure of R and S chiral ferroelectric crystals. b) XRD patterns of R/S chiral ferroelectric crystals. c) Circular dichroism spectra of R/S chiral ferroelectric films. The insert is the crystal structures of R (upper) and S (lower) chiral molecular ferroelectrics at room temperature. d) The variation of SHG intensities in R chiral ferroelectric thin films under different polarization states of light excitation. e) Schematic diagram of the coupling effect between ferroelectric order, electric field, and CPL photons.

Chiral molecules can enhance the likelihood of forming polar structures, while organometallic ligands can elevate the potential energy barriers of molecular dynamics, thereby increasing its Curie temperature. Therefore, the combination of chiral molecules and organometallic ligands is an effective approach to achieving room‐temperature chiral ferroelectricity. More importantly, the integration of axial chirality and ferroelectricity provides an ideal platform for enhancing the coupling between CPL photons and electric dipoles. As illustrated in **Figure** **1a**, the combination of organosilicon compounds with chiral molecules (R)−1,1′‐bi(2,2′‐naphthol) [(R)‐(+)‐BINOL] and (S)−1,1′‐bi(2,2′‐naphthol) [(S)‐(‐)‐BINOL] results in a stable, axially chiral molecular ferroelectric structure. The 1,1′‐bi‐2,2′‐naphthol (BINOL) family is commonly used in the preparation of organic chiral materials, while organosilicon ligands are known for their non‐toxic, flexible, and environmentally friendly properties. Consequently, the chiral molecular ferroelectric materials synthesized from this combination hold significant promise in eco‐friendly flexible ferroelectrics for biomedical fields. The formation of the chiral ferroelectric crystal is due to the bonding between chiral molecules and organosilicon component, resulting in an asymmetric structure that induces a dipole moment. The presence of O‐H bonds within the R and S chiral precursor molecules results in two vibrational absorption peaks within the 3450–3550 cm⁻¹ range (Figure S1, Supporting Information). However, these O─H bond vibrational peaks are absent in the pure R and S chiral ferroelectric crystal. This indicates that the chiral small molecules form bonds with organosilicon component, leading to the disappearance of the O─H bonds. The XRD patterns of the R and S chiral ferroelectric films have identical diffraction peak positions (Figure 1b). This indicates that they belong to the same crystal system. Moreover, the EDS mapping of the R and S chiral ferroelectric crystals shows the presence of both oxygen and chlorine elements (Figures S2 and S3, Supporting Information). This indicates that the chiral molecular precursors have bonded with the organosilicon component and crystallized. The R/S chiral ferroelectric crystals are transparent, with their absorption spectrum mainly concentrated in the UV wavelength range (Figure 1b; Figure S4, Supporting Information). At room temperature, the circular dichroism (CD) spectra of chiral R/S ferroelectric films exhibit a pronounced CD signal near 349 nm, shown in Figure 1c. Moreover, the CD signals of the R and S ferroelectric films are mirror images of each other. This mirror‐image symmetry in the CD spectra indicates the enantiomeric nature of such films. The origin of the CD signal in chiral materials can be attributed to the differential absorption of LCP and RCP. Consequently, this observation suggests that the absorption coefficients for LCP and RCP are opposite for the R/S chiral ferroelectric films. Specifically, the R chiral ferroelectric film exhibits a greater absorption coefficient for LCP, while the S chiral ferroelectric film shows a higher absorption for right‐handed circularly polarized photons. The differential absorption of LCP and RCP offers the potential for CPL to modulate the polarization of chiral ferroelectrics. As illustrated in Figure 1d and Figure S5 (Supporting Information), with the rotation angle of the quarter‐wave plate, the polarization type of the excitation light transitions from linearly polarized light to RCP when the angle between the fast axis of the wave plate and the direction of linearly polarized light is 45°. Further rotation to 135°converts the light to LCP. There is a significant dependence between the second harmonic generation (SHG) signal intensity of chiral ferroelectric thin films under the polarization type of the excitation light (RCP vs LCP). The SHG signal intensity of the chiral ferroelectric thin films reaches its maximum under RCP excitation, whereas it is minimized under LCP excitation. The intensity of the SHG signal is proportional to the square of the polarization strength of the ferroelectric material. This indicates that the polarization of chiral ferroelectric thin films can be effectively regulated by the polarization state of CPL. Additionally, the difference in ferroelectric polarization strength is maximized when switching between LCP and RCP. This chiral ferroelectric material, which possesses both chirality and ferroelectricity, enables the coupling between CPL photons and electric dipoles (Figure 1e). Additionally, it achieves the non‐contact modulation of the polarization in ferroelectric crystals using CPL. This provides a potential pathway for the development of future non‐contact, fast‐response CPL sensing devices.

**Figure 2:**
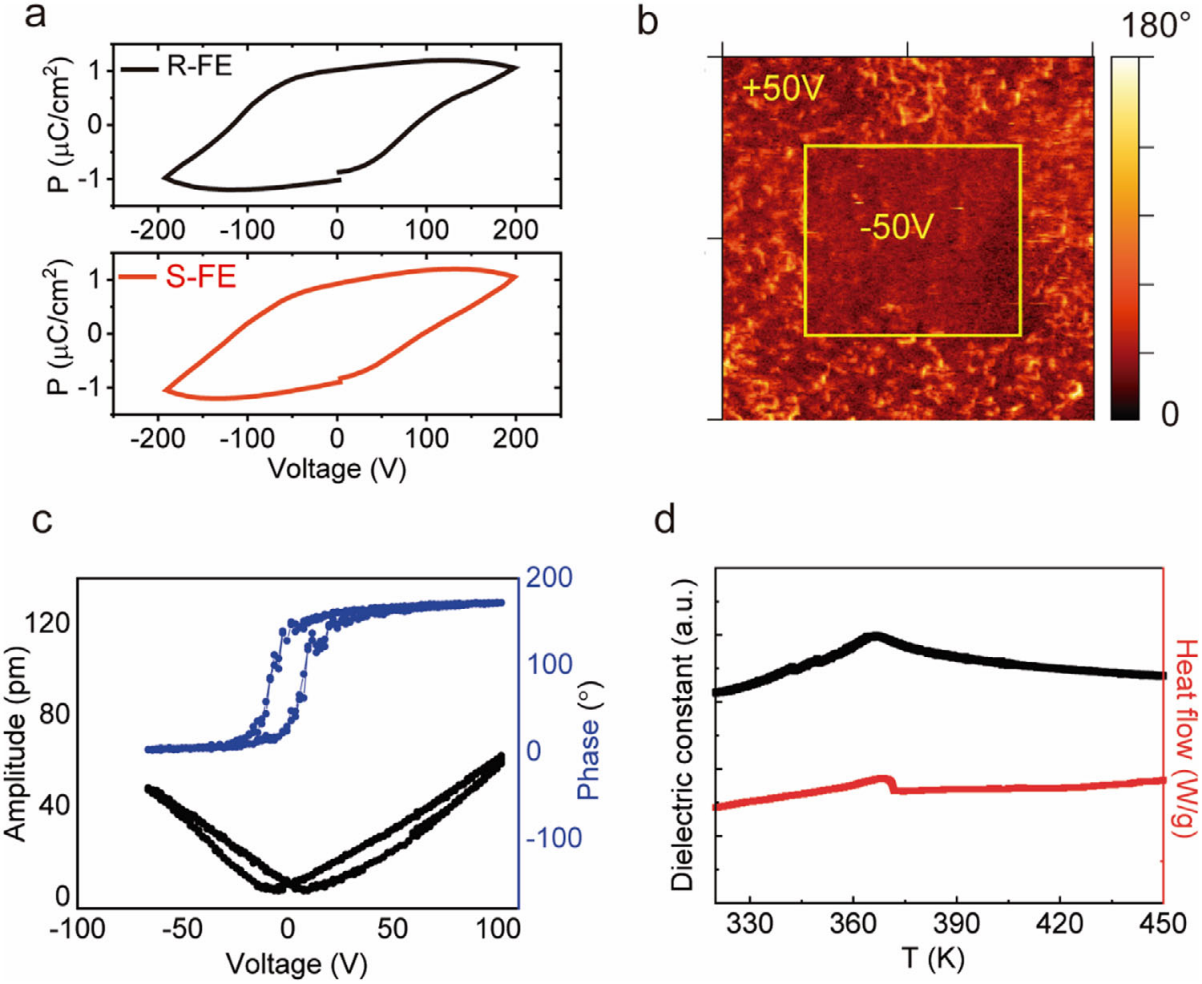
a) The P‐E hysteresis loop of R/S chiral ferroelectric films. b) PFM phase images mapped phase‐switching of R chiral ferroelectric films. c) PFM amplitude (black) and phase (blue) hysteresis loops of R chiral ferroelectric thin film. d) Temperature dependent dielectric constants (black) and DSC curves (red) of R chiral ferroelectric thin films.

As illustrated in **Figure** **2a**, the chiral films exhibit ferroelectricity at room temperature. Both the R and S chiral ferroelectric films demonstrate a saturation polarization value of ≈1.2 µC cm^−^^2^. To further confirm its ferroelectricity, we utilize the piezoresponse force microscopy (PFM) images and PFM hysteresis loops to study ferroelectric domains and local ferroelectric polarization switching behavior. As shown in Figure 2b,c and Figure S6 (Supporting Information), distinct ferroelectric domains and PFM hysteresis were clearly observed in the chiral ferroelectric films. The polarization switching and hysteresis characteristics in the ferroelectric domains and square phase loops indicate the ferroelectricity of the chiral ferroelectric films. To more intuitively observe the ferroelectric domain flipping process in the chiral ferroelectric films, we performed local polarization writing tests on the film surface. We applied +50 V and −50 V DC tip biases to scan the central region of the chiral ferroelectric films. The enhanced PFM amplitude signal was obtained in the electrically biased regions (Figure 2b). Significant differences could be observed in the oppositely polarized regions, with clear distinctions between bright and dark areas. In the corresponding PFM phase images, the bright and dark areas correspond to upward and downward polarization states, respectively, exhibiting significant phase contrast. The appearance of hysteresis in the PFM signal indicates the presence of ferroelectricity (Figure 2c). As shown in Figure 2d, the dielectric constant of the R/S chiral ferroelectric films, measured at a frequency of 1000 Hz, exhibits a sharp peak ≈363 K as the temperature increases. This indicates a phase transition from ferroelectric to paraelectric state near 363 K in the chiral ferroelectric films. The obvious anomaly in the dielectric constant as a function of temperature is attributed to the first‐order structural phase transition in the chiral ferroelectric films. This transition is caused by the order‐disorder transition of the isopropyl groups in the organosilicon ligands within the chiral ferroelectric films, leading to the ferroelectric‐paraelectric phase transition. Furthermore, according to the DSC curve during the heating process, an exothermic peak is observed ≈363 K. Additionally, the crystal structure of the chiral ferroelectric crystals changes from the original triclinic P1 system to the monoclinic P21 system.

**Figure 3:**
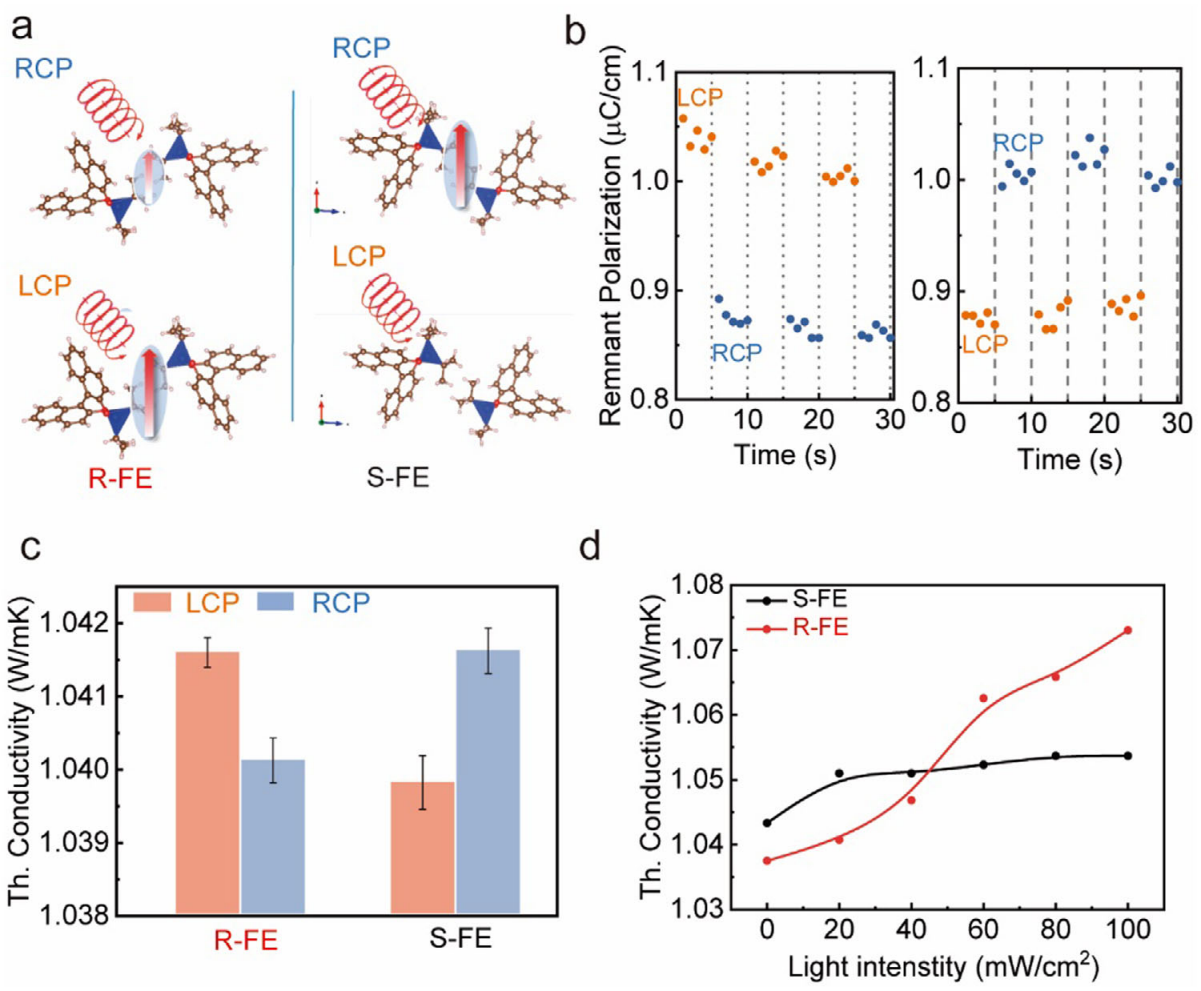
a) A schematic diagram the polarization tuned by CPL in chiral ferroelectric crystals. The red arrow represents the dipole moment, and its length indicates the magnitude of the total dipole moment. b) Remnant polarization of R (left) and S (right) chiral ferroelectric thin films under LCP and RCP excitation. c) Thermal conductivity of R and S chiral ferroelectric crystals under LCP and RCP excitation. d) The variation in thermal conductivity of R and S chiral ferroelectric crystals with increasing intensity of CPL.

Chiral ferroelectrics, which exhibit both axial chirality and ferroelectricity, offer the potential for polarization control using CPL. **Figure** **3a** illustrates the polarization of electric dipoles in chiral ferroelectric thin films induced by CPL. In R/S chiral ferroelectric thin films, we can regulate the polarization by switching the type of circular polarization. Figure 3b illustrates the remnant polarization of R chiral ferroelectric thin films under LCP and RCP excitation. The remnant polarization is ≈1.05 µC cm^−2^ under LCP excitation. However, when the excitation light is switched to RCP, the remnant polarization significantly decreases to ≈0.85 µC cm^−2^. In contrast, for S chiral ferroelectric thin films, the opposite phenomenon is observed. The remnant polarization under RCP excitation is ≈0.98 µC cm^−2^, which is significantly higher than the 0.85 µC cm^−2^ observed under LCP excitation. Photogenerated charges play a crucial role in the interaction between ferroelectric polarization and light in ferroelectric materials. On one hand, the photoelectric effect in ferroelectric materials can induce electrostrictive or piezoelectric effects, causing lattice deformation and thus regulating polarization. On the other hand, ferroelectric materials and electrodes can generate photogenerated charges through photon absorption, which accumulate at the interface, creating an electrostatic field effect. This electrostatic field induced by the accumulation of photogenerated charges can modulate the polarization field within ferroelectric materials, thereby controlling its ferroelectric polarization. Chiral ferroelectric crystals exhibit both ferroelectricity and ferroelasticity, with electrodes playing a role during testing. In ferroelectric materials, ferroelectric polarization arises from ionic displacement, leading to concomitant changes in the lattice structure. Consequently, modifications in ferroelectric polarization condition the electron‐phonon interactions within ferroelectric materials, thereby inducing variations in thermal conductivity. This relationship is rooted in the strong correlation between phonon transport and ferroelectric polarization. As illustrated in Figure 3c, for R chiral ferroelectric thin films, the greater remnant polarization induced by LCP, compared to RCP, results in an increase in thermal conductivity. Conversely, S chiral ferroelectric thin films exhibit the opposite behavior, with a thermal conductivity of ≈1.04 W mK^−1^ under LCP, which is lower than the 1.042 W mK^−1^ observed under RCP. This phenomenon can be attributed to the fact that enhanced ferroelectric polarization in these materials reduces phonon scattering, thereby increasing the phonon mean free path within the ferroelectric crystal.^[^ ^40^, ^41^ ^]^ Since thermal conductivity is directly proportional to the phonon mean free path, the thermal conductivity of chiral ferroelectric crystals increases with enhanced ferroelectric polarization.^[^ ^42^, ^43^ ^]^ The observed variations in thermal conductivity under LCP and RCP align with the corresponding changes in remnant polarization. Additionally, as the excitation intensity of left‐ or RCP is further increased from 10 to 100 mW cm^−^^2^, ferroelectric polarization is further enhanced, leading to a corresponding increase in thermal conductivity from ≈1.04 to 1.07 W mK^−1^. This further corroborates the role of CPL in regulating ferroelectric polarization in chiral ferroelectric thin films.

**Figure 4:**
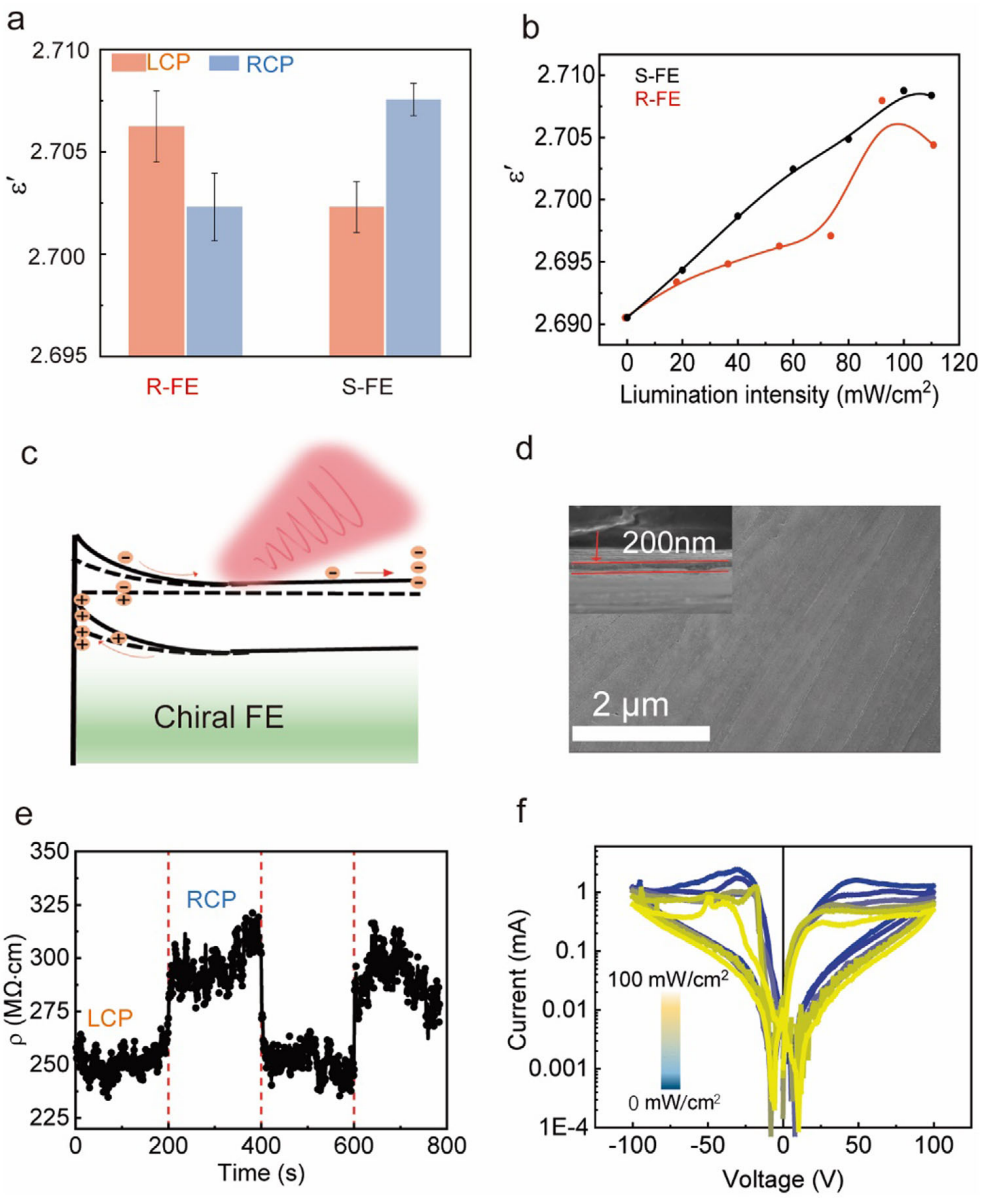
a) Dielectric constant variation with frequency of R (left) and S (right) chiral ferroelectric thin films under LCP and RCP light excitation at 1000 Hz. b) The dielectric constant of R and S chiral ferroelectric crystals under varying intensities of CPL excitation. c) Schematic diagram of photogenerated charges and charge accumulation in chiral ferroelectric thin films. d) The SEM image of R chiral ferroelectric thin films. The insert shows a cross‐sectional image of the R chiral ferroelectric thin film. e) Resistance changes of chiral ferroelectric thin films under LCP and RCP excitation. f) Light intensity dependent IV curves of chiral ferroelectric films.

To further confirm the origin of light‐regulated polarization in chiral ferroelectrics, we measured the frequency‐dependent dielectric constant under LCP and RCP excitation. As shown in **Figure** **4a**, for R chiral ferroelectrics, the dielectric constant under LCP excitation is greater than that of under RCP excitation at both low and high frequencies. The dielectric constant at low frequencies corresponds to space charges, while at high frequencies it corresponds to dipoles (Figures S7 and S8, Supporting Information). This indicates that illumination not only generates photogenerated free charges but may also induce intramolecular charge transfer within chiral ferroelectric molecules. Moreover, as the illumination intensity increases, the dielectric constant of the chiral ferroelectric thin film progressively rises. This suggests that with the increase in excitation light intensity, more photo‐generated charges are produced within the chiral ferroelectric thin film (Figure 4b). To further verify the regulation of polarization by light in chiral ferroelectric thin films, we investigated light dependent current density and resistance under LCP and RCP excitation using R chiral ferroelectric thin films. As shown in Figure 4c, the interface between the chiral ferroelectric thin film and electrode exhibits a Schottky‐like band bending effect due to charge redistribution. This band bending causes photogenerated carriers to move directionally due to the potential difference, accumulating at the interface. As shown in Figure 4d and Figure S9 (Supporting Information) with the R‐chiral ferroelectric thin films with an average thickness of ≈200 nm. This uniformity is conducive to the efficient transport of free charges under the influence of an electric field. The thin film and the band bending at the interface induced a tunneling effect of photogenerated charges, resulting in changes in current density and resistance. According to Figure S10 (Supporting Information), the current of the R chiral ferroelectric thin film under LCP excitation is ≈5 mA, higher than the current under RCP excitation (≈2 mA). Correspondingly, the resistance is also significantly lower under LCP excitation (Figure 4e). This may be due to the greater absorption of photogenerated carriers by LCP, leading to further band bending at the interface, increased tunneling electrons, and thus higher current and lower resistance, with resistance decreasing from 300 to ≈250 MΩ·cm. This results in an increase in polarization in the chiral film due to the electrostatic field, thereby leading to an increase in resistance. This process is reversible and consistent with the results of polarization regulation by CPL. As the illumination intensity increases, more photogenerated charges accumulate at the interface, causing further band bending and enhancement of the built‐in electric field. Consequently, the polarization is further enhanced. Therefore, as the illumination intensity increases from 10 to 50 mW cm^−^^2^, the current exhibits a decreasing trend (Figure 4f). The CPL modulation, achieved by switching between LCP and RCP, offers a novel and precise approach to controlling the polarization of chiral organic ferroelectric molecules, presenting new potential for applications related to ferroelectric polarization. Compared to thermally induced polarization modulation via photothermal effects, which is often restricted to shallow surface layers due to severe temperature gradients, CPL‐controlled polarization demonstrates unique advantages. It not only enables polarization modulation through photoinduced non‐equilibrium carriers but also allows direct modulation by altering the type of polarized light. This introduces a new degree of control and achieves higher resolution, significantly enhancing the flexibility and practicality of CPL‐controlled polarization for ferroelectric devices. Furthermore, chiral organic ferroelectric materials can be fabricated into devices via solution‐based processing, offering a simpler, more cost‐effective, and industrially scalable method. These materials also exhibit flexibility, making them particularly suitable for flexible electronics applications. Despite its tremendous potential, CPL‐controlled polarization remains in its early stages of development. A key challenge is the instability of polarization switching induced by polarized light, as polarization often reverts to its initial state upon the removal of the light, limiting its application in non‐volatile memory devices. Additionally, due to bandgap constraints, the absorption spectrum of chiral organic ferroelectrics is primarily concentrated in the UV region. Compared to narrow‐bandgap inorganic 2D ferroelectric materials, the shorter writing wavelengths required for chiral organic ferroelectrics present a notable limitation. To address these challenges, future research should focus on developing chiral organic ferroelectric or chiral 2D ferroelectric materials with narrower bandgaps to enable broader writing wavelength ranges and higher stability in polarization switching. Improving the stability of CPL‐induced polarization switching will be critical to laying the foundation for next‐generation applications in non‐volatile memory and electro‐optic devices. These innovations are expected to provide new directions for the development of ferroelectric devices.

## 3 Conclusion

In this study, we demonstrated the coupling effect between circularly polarized photons and electric dipoles based on chiral ferroelectrics. Due to the asymmetric chiral structure, chiral ferroelectrics exhibit distinct absorption coefficients for LCP and RCP, resulting in the generation of different amounts of photogenerated charges. The electrostatic field generated by the accumulation of these photogenerated charges at the interface effectively enhances the polarization of chiral ferroelectric thin films. When switching from LCP to RCP, the remanent polarization of the R‐chiral ferroelectric thin film increases from 0.85 to 1.05 µC cm^−^^2^, an approximately fourfold increase. Conversely, for the S‐chiral ferroelectric thin film, the remanent polarization decreases by ≈23%. Furthermore, the reversal of polarization in the ferroelectric thin film enables the reversible control of current by CPL. Thus, we successfully achieved ferroelectric polarization modulation using CPL in organic axial chiral molecular ferroelectrics through the generation of photoinduced non‐equilibrium carriers. This approach enables polarization control not only by adjusting light intensity but also by altering the type of polarization. This dual capability effectively overcomes the limitations of traditional electric field‐ and strain‐induced polarization control methods and provides a novel pathway and additional degrees of freedom for non‐contact, remote, and ultrafast optical control of polarization in ferroelectric devices. The unique ability of CPL‐induced polarization control to introduce new degrees of freedom lays a solid foundation for developing more versatile and high‐performance ferroelectric optoelectronic devices. Additionally, the solution‐processability and inherent flexibility of organic chiral molecular ferroelectrics make them particularly promising for the development of next‐generation high‐speed, non‐contact, low‐cost non‐volatile memory devices and flexible ferroelectric optoelectronic devices based on CPL control.

## 4 Experimental Section

### Synthesis of Chiral Crystals

A mixture of 1,1′‐bi‐2‐naphthol (4.0 g), anhydrous triethylamine (7.7 mL), and DCM (100 mL) was placed in a round‐bottom flask. Dichlorodiisopropylsilane (2.6 g) was added dropwise, and the reaction was stirred overnight at room temperature. After adding water (100 mL), the organic phase was washed with saturated NaHCO₃ solution three times (3 × 50 mL), dried with Na₂SO₄, and concentrated. The product recrystallized from ethyl acetate. The samples crystallized from ethyl acetate were washed with isopropanol (IPA) to remove the uncrystallized impurities, resulting in colorless, transparent crystals.

### Preparation of Chiral Crystal Thin Films

Precursor solutions of R‐FE and S‐FE were prepared by dissolving 20 mg of the chiral crystals in 0.4 mL of tetrahydrofuran. The solutions were then spin‐coated onto indium tin oxide (ITO) glass substrates at a rotation speed of 6000 rpm. The resulting thin films were annealed at room temperature for 2 h to complete the process.

### Measurement Methods

The polarization‐electric field (P‐E) hysteresis loops were measured using a Precision LC Ferroelectric Tester, equipped with a high‐voltage interface and a Trek 609B high‐voltage amplifier (Radiant Technologies Inc., USA). This setup enabled precise control and analysis of the polarization behavior in the sample. Temperature‐dependent dielectric constant measurements at 10 kHz were performed using an Agilent 4294A impedance analyzer and a box furnace. Silver epoxy electrodes were applied to the specimens for both P‐E hysteresis and dielectric measurements. Piezoresponse force microscopy (PFM) was conducted using an Oxford Instruments Cypher ES microscope with a high‐voltage package and in situ heating. Conductive Pt/Ir‐coated silicon probes (EFM, Nanoworld) with a nominal spring constant of ≈2.8 nN nm^−1^ and a resonance frequency of ≈75 kHz were used for domain imaging and polarization switching. Due to the low amplitude of vertical PFM signals, measurements were conducted at the contact resonance to minimize noise interference from the AFM's photodetector. Second harmonic generation (SHG) measurements were conducted using a lens pair to collect reflected light, which was transmitted via fiber to an HRS‐300 spectrometer with a PyLoN camera system (Princeton Instruments). The seed laser was generated by an Astrella‐F‐1K femtosecond amplifier (Coherent), operated at 1 kHz and the tunable wavelength were generated by optical parametric amplifier from Ultrafast System, with the intensity‐controlled with a variable ND filter. Excitation was done with an 850 nm long‐pass filter and a focusing lens. Polarized SHG was achieved using a linear polarizer and a quarter‐wave plate placed before the sample. Thermal analysis was performed using a DSC 7 differential scanning calorimeter (Perkin Elmer) at a heating rate of 5 °C min^−1^, and thermal degradation was assessed using a thermogravimetric analyzer (TGA) (SDT Q600, TA Instruments) under nitrogen. Powder X‐ray diffraction (PXRD) data were measured using a Bruker D8 Advance X‐ray diffraction system with Cu Kα radiation with a step size of 0.1°. The microstructural morphology and elemental distribution of the chiral ferroelectric crystals were analyzed using scanning electron microscopy (Hitachi SU‐70 FEG SEM) combined with energy‐dispersive X‐ray spectroscopy (EDS) mapping. Fourier‐transform infrared spectroscopy (FTIR, Agilent Cary 560) was employed to investigate the chemical bonding characteristics within the chiral ferroelectric crystals.

## Conflict of Interest

The authors declare no conflict of interest.

## Supporting information



Supporting Information

## Data Availability

The data that support the findings of this study are available from the corresponding author upon reasonable request.
