# Supplementary material for: Reversible Ferroelectric Polarization Modulation of Chiral Molecular Ferroelectrics by Circularly Polarized Light
Source: Adv Sci (Weinh). 2025 Jan 21;12(10):2414977. doi: 10.1002/advs.202414977 (PMC11905069; doi:10.1002/advs.202414977)
Supplement: Supplementary file 1 — Supporting Information [file ADVS-12-2414977-s001.docx]

Supporting Information

**Reversible ferroelectric polarization modulation of chiral molecular ferroelectrics by circularly polarized light**

*Zhongxuan Wang^[a]^, Qian Wang^[b]^, Lina Quan^[b], [c]^, Shenqiang Ren* ^[a]^*

^[a]^ Z. X. Wang, S. Q. Ren

Department of Materials Science and Engineering

University of Maryland

College Park, MD, 20742, USA

sren@umd.edu

^[b]^ Q. Wang, L. Quan

Department of Chemistry

Virginia Tech

Blacksburg, Blacksburg, Virginia 24060, USA

^[c]^ L. Quan

Department of Materials and Science Engineering

Virginia Tech

Blacksburg, Blacksburg, Virginia 24060, USA


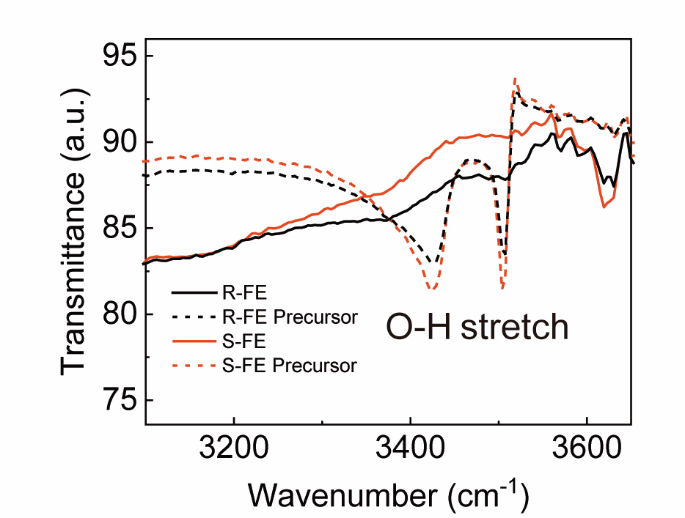


Figure S1. FTIR spectra of R/S chiral ferroelectric crystals and their precursors.


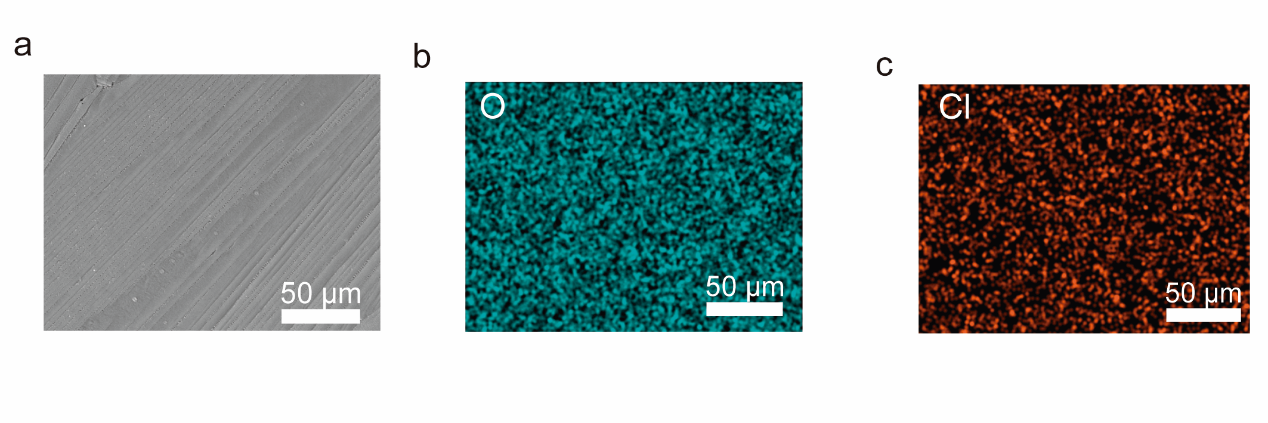


Figure S2. The EDS mapping image of R chiral ferroelectric crystal films.


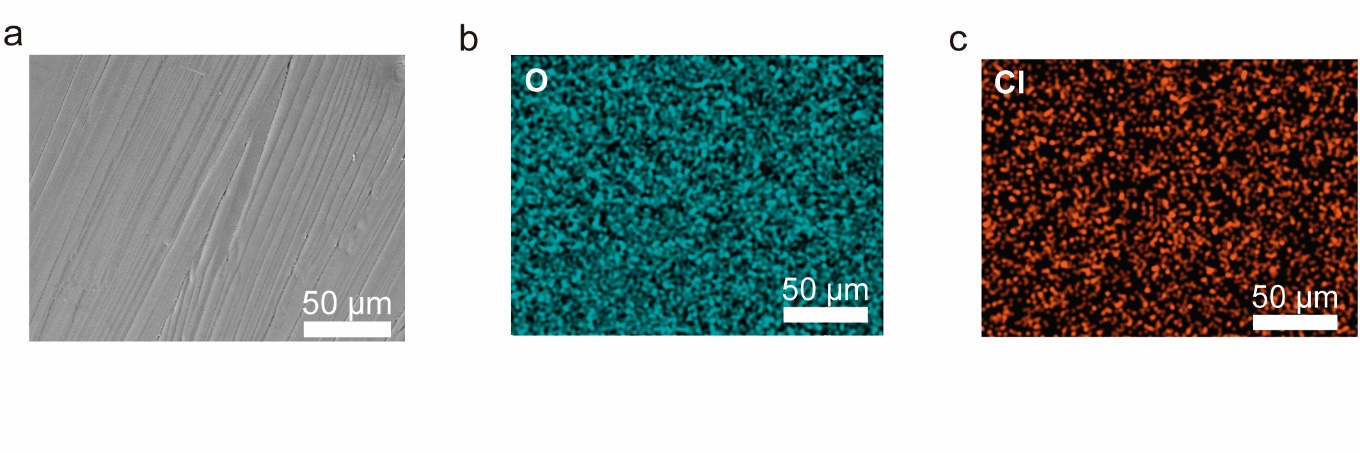


Figure S3. The EDS mapping image of S chiral ferroelectric crystal films.


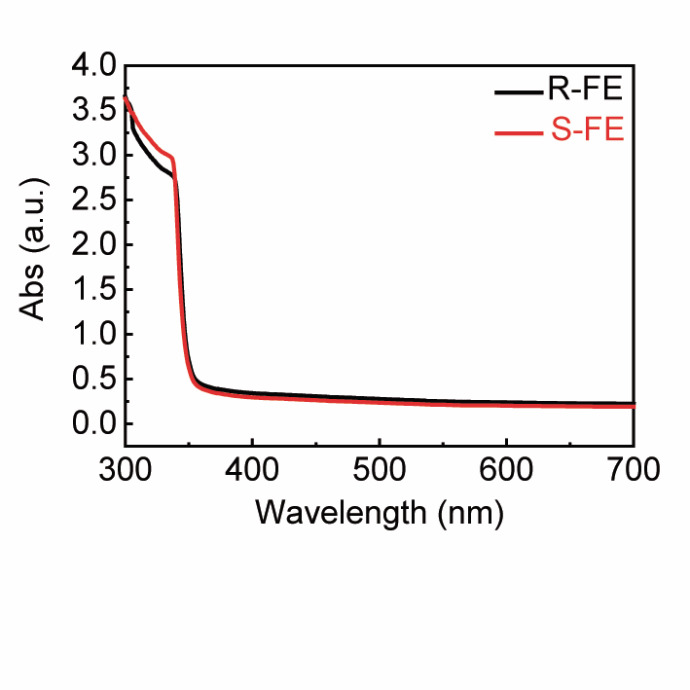


Figure S4. The Absorption spectrum of chiral ferroelectric crystal films.


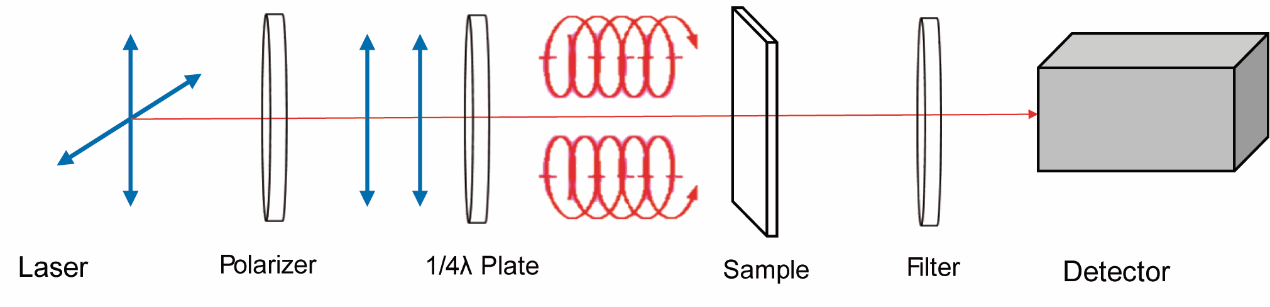


Figure S5. Schematic diagram of the SHG signal testing apparatus under circularly polarized light excitation.


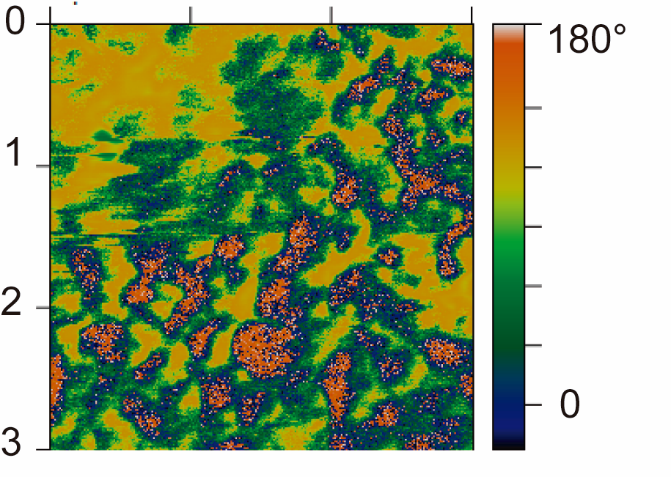


Figure S6. The phase images of R chiral ferroelectric films.


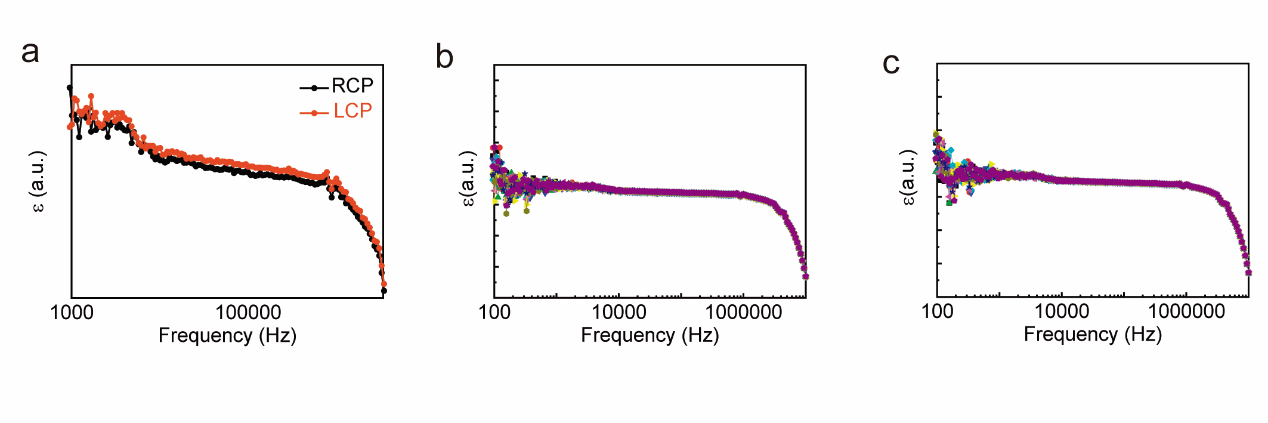


Figure S7. The frequency dependent dielectric constant curves of R chiral ferroelectric films under left (b) and right (c) circularly polarized light excitation.


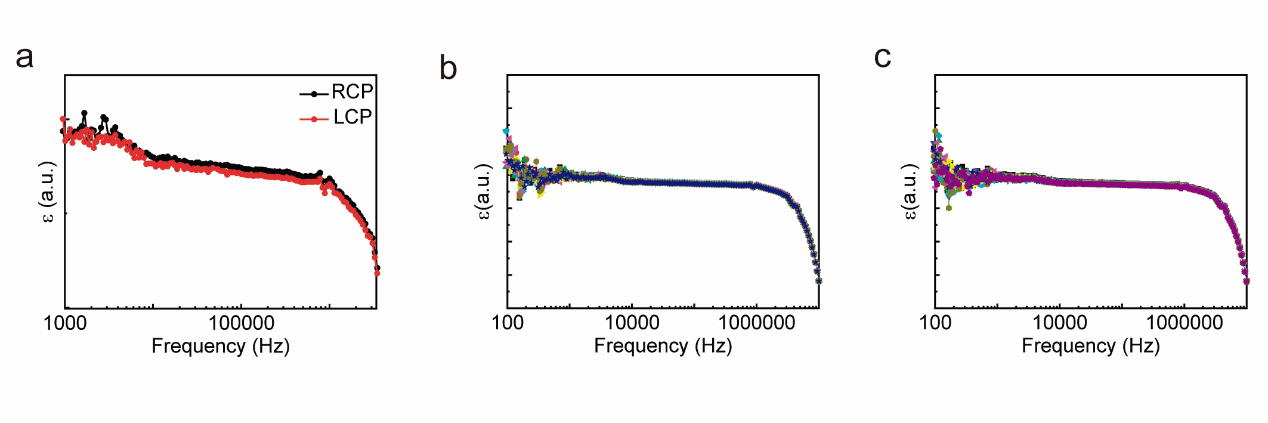


Figure S8. The frequency dependent dielectric constant curves of S chiral ferroelectric films under left (b) and right (c) circularly polarized light excitation.


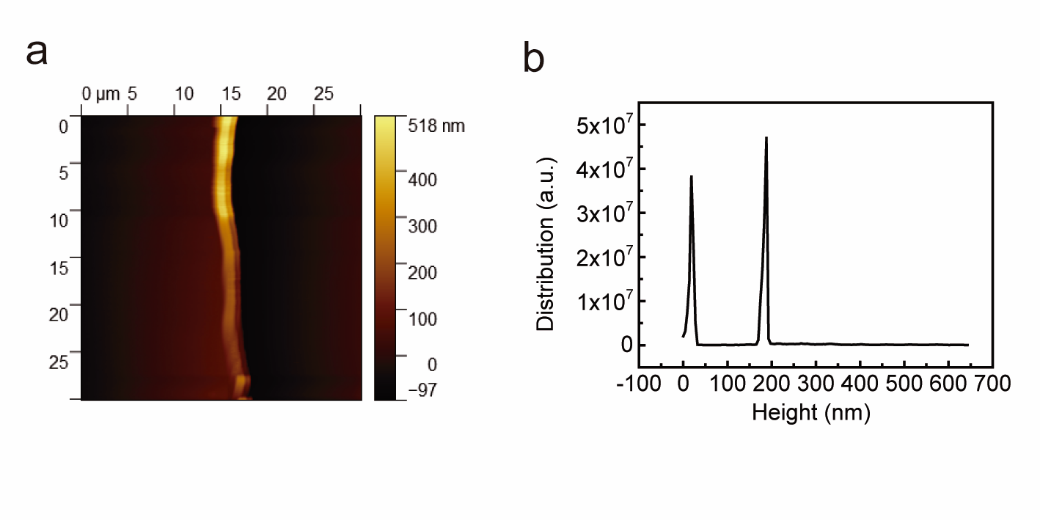


Figure S9. The AFM images and height distribution statistics of R-chiral ferroelectric films.


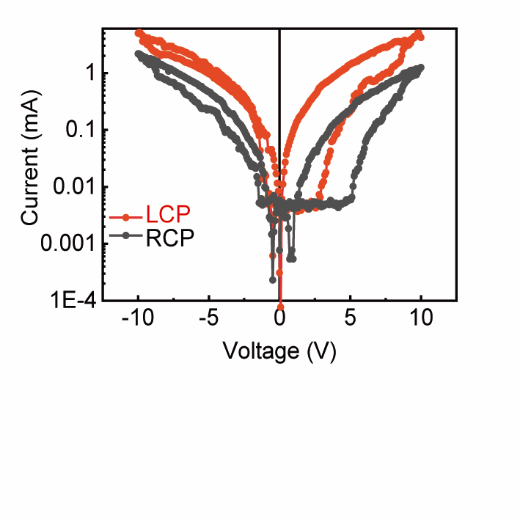


Figure S10. The IV curve of R-chiral ferroelectric films under left and right circularly polarized light excitation.
